# Advances in Understanding Renin–Angiotensin System-Mediated Anti-Tumor Activity of Natural Polyphenols

**DOI:** 10.3390/biom15111541

**Published:** 2025-11-02

**Authors:** Ximing Wu, Mingchuan Yang, Hailing Zhang, Lumin Yang, Yufeng He, Xiaozhong Cheng, Guilan Zhu

**Affiliations:** 1 Anhui Province Green Food Collaborative Technology Service Center for Rural Revitalization, School of Biological and Food Engineering, Hefei Normal University, Hefei 230601, China; wxm2022@hfnu.edu.cn (X.W.); zhl@hfnu.edu.cn (H.Z.); 2 Tea Research Institute, Chinese Academy of Agricultural Sciences, Hangzhou 310008, China; yangmingchuan@tricaas.com; 3 Key Laboratory of Biodiversity Conservation and Characteristic Resource Utilization in Southwest Anhui, School of Life Sciences, Anqing Normal University, North Jixian Road, No. 1318, Anqing 246133, China; lumin@aqnu.edu.cn; 4 State Key Laboratory of Tea Plant Germplasm Innovation and Resource Utilization, Anhui Agricultural University, Hefei 230036, China; qhyf2019@ahau.edu.cn

**Keywords:** renin–angiotensin system, polyphenols, anti-tumor activity, thiol-containing transmembrane proteins, RAS dual-axis regulation

## Abstract

The imbalance of the renin–angiotensin system (RAS), characterized by the overactivation of the pro-tumor ACE/AngII/AT1R axis, is closely linked to tumor growth, angiogenesis, metastasis, and poor prognosis. Natural polyphenols, such as EGCG and resveratrol, exert anti-cancer effects by dual-regulating RAS: they inhibit the pro-tumor axis by blocking renin, ACE activity, and AT1R expression, while simultaneously activating the protective ACE2/Ang(1-7)/MasR axis. Furthermore, polyphenols and their autoxidation products (e.g., EAOP) modify thiol-containing transmembrane proteins (such as ADAM17 and integrins) and interact with RAS components, further disrupting oncogenic pathways (including MAPK and PI3K/Akt/mTOR) to induce apoptosis, suppress invasion, and reduce oxidative stress. Notably, EAOP exhibits stronger RAS-modulating efficacy than its parent polyphenols. However, challenges such as low bioavailability, insufficient targeting, and limited clinical evidence impede their application. This review provides a comprehensive overview of the anti-cancer mechanisms of polyphenols through RAS regulation, discusses the associated challenges, and proposes potential solutions (including nanodelivery and structural modification) and strategies to advance natural product-based adjuvant treatments.

## 1. Introduction

The renin–angiotensin system (RAS) comprises various interconnected elements. Under normal physiological conditions, components of RAS—including angiotensin II (AngII), its receptors (AT1R and AT2R), and downstream effectors—regulate cellular activities such as vasoconstriction and the maintenance of electrolyte balance [1,2,3]. RAS is present in both healthy and malignant tissues. However, in cancerous tissues, the regulation of RAS components is significantly disrupted, resulting in their heightened expression. For instance, in clear cell renal cell carcinoma (CC-RCC), there is a notable increase in the levels of renin and angiotensin-converting enzyme (ACE), a phenomenon also observed in other cancers such as reninoma, where renin levels exceed those found in normal tissues [4,5]. Additionally, the (Pro)renin receptor [(P)RR] exhibits abnormal elevation in pancreatic and colorectal cancers, contributing to tumor development and progression [6,7,8]. The irregular expression of RAS has been linked to the initiation and advancement of various cancers. In CC-RCC, the overexpression of ACE correlates with unfavorable outcomes and influences tumor invasion and metastasis [5]. In glioblastomas, the expression of RAS-related genes has been associated with reduced survival rates for patients [9]. Furthermore, in pancreatic ductal adenocarcinoma and colorectal cancer, the abnormal expression of (P)RR fosters tumorigenesis by causing genomic instability and disrupting signaling pathways [6,7,10], underscoring the critical role of RAS in cancer biology. Clinical evidence indicates that RAS inhibitors (RASi), such as ACE inhibitors, can impede tumor progression, further highlighting the detrimental role of RAS in cancer [2,11]. The dysregulation of RAS is intricately linked to cancer development and progression, primarily affecting the tumor microenvironment, angiogenesis, invasion, survival signaling, and proliferation, as discussed in Section 2 of the article. Targeting the RAS axis offers a promising strategy for cancer treatment, particularly by modulating RAS components that exhibit diverse effects.

Plant-derived polyphenols, such as epigallocatechin gallate (EGCG) from tea, tannic acid, caffeic acid (found in fruits and coffee), resveratrol (derived from grapes and berries), and chlorogenic acid (extracted from honeysuckle), exhibit significant anti-cancer properties in preclinical studies by modulating key oncogenic signaling pathways [12,13,14,15,16,17]. In various cellular models, these compounds demonstrate a spectrum of anti-cancer activities: EGCG and quercetin diminish QSOX1 secretion and alter its localization in liver cancer cells (HepG2, Huh7) [12]; carbon nanodots derived from caffeic acid inhibit the KRAS/BRAF/MEK/ERK signaling cascade [18]; hydroxycinnamic acids, including caffeic acid, influence the biochemical properties of HepG2 cells similarly to pravastatin [19]; and polyphenols (EGCG, tannic acid, curcumin) can reverse multidrug resistance by inhibiting P-glycoprotein (P-gp) in resistant cell lines (CEM/ADR 5000, CaCo2) [20]. However, achieving effective cytotoxicity often requires high micromolar concentrations, raising concerns about potential genotoxic effects on healthy cells [21]. Animal studies support their efficacy, with caffeic acid (5.4 mg/kg) enhancing the tumor-suppressive effects of doxorubicin by 85.6% in xenograft models [22] and dietary polyphenols (chlorogenic acid, EGCG, resveratrol) reducing arsenic bioavailability and toxicity, thereby potentially decreasing cancer risk [14].

The mechanisms through which these compounds exert their anti-tumor effects include the inhibition of critical pathways such as mitogen-activated protein kinase (MAPK) (KRAS/BRAF/MEK/ERK) [18], PI3K/Akt/mTOR (which regulate growth, metabolism, and apoptosis) [23,24], NF-kappaB (NF-κB), and signal transducers and activators of transcription (STATs) (which mitigate inflammation and disease progression) [25], along with the inhibition of P-gp [20]. These combined actions lead to apoptosis [26], cell cycle arrest, reduced invasion and metastasis [25,27], and modulation of oxidative stress, acting as pro-oxidants in tumors and antioxidants in normal tissues [28,29], often working synergistically with chemotherapy [20,22,30,31]. Despite their potential, polyphenols face significant challenges. Their low water solubility and short half-life contribute to poor bioavailability, limiting their effectiveness in vivo and often necessitating high doses that could be detrimental to normal cells [21,30,32,33]. Targeted delivery remains problematic, as polyphenols do not specifically target cancer cells [30]; innovations such as nanodelivery systems are being developed to enhance localized release and minimize off-target effects [34,35]. Additionally, their mechanisms are intricate and non-specific, affecting multiple molecular targets without selectivity. This lack of specificity complicates their therapeutic use, as polyphenols can influence various pathways simultaneously, including redox balance, cytokine production, and epigenetic changes, raising the likelihood of unintended consequences and limiting dose optimization [26,28,36,37]. In conclusion, while polyphenols present a promising avenue as adjunctive anti-cancer agents, it is crucial to address issues related to bioavailability, delivery methods, and the complexity of their mechanisms to fully realize their clinical potential.

Beyond their ability to combat tumors, natural polyphenols act as multifaceted modulators of RAS, influencing both the classical and alternative RAS axes [38,39]. For instance, polyphenols and their derivatives, including flavonoids, have been shown to inhibit renin activity. Research utilizing molecular docking techniques has demonstrated that flavonoids such as luteolin, quercetin, and kaempferol can interact with human renin (PDB: 2V0Z), resulting in an inhibitory effect comparable to that of Aliquilan [40]. Additional insights into the influence of polyphenols on RAS are discussed in Section 3.2 of this article. Given the significant link between RAS and cancer progression, along with the regulatory effects of natural polyphenols on RAS, this review investigates the potential of natural polyphenols and their derivatives for anti-tumor applications through RAS modulation, particularly as adjuvant therapies in cancer therapies. Furthermore, it addresses challenges related to the low bioavailability of polyphenols in cancer therapy and the necessity for targeted delivery to tumors. In summary, this review provides a novel perspective on the development of anti-cancer drugs derived from natural polyphenols that focus on RAS regulation.

## 2. The Impact of RAS on Cancer

### 2.1. The Dysregulation of RAS Components in Cancer

**Figure 1:**
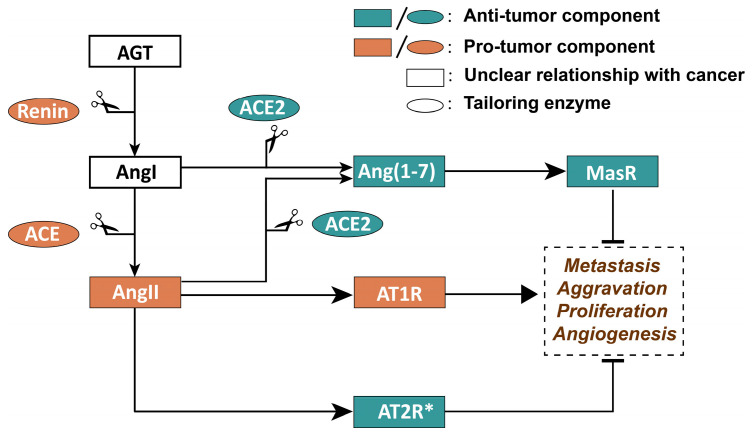
Schematic of the renin–angiotensin system (RAS) dual-axis and its association with tumor progression. Component definitions: AGT (angiotensinogen): RAS precursor, cleaved by renin to form AngI. Renin: catalyzes AGT → AngI; upregulated in renal cell carcinoma [4,5]. ACE (angiotensin-converting enzyme): catalyzes AngI → AngII (pro-tumor axis); overexpressed in gastric cancer [45]. ACE2 (angiotensin-converting enzyme 2): catalyzes AngII → Ang(1-7) (anti-tumor axis); downregulated in gallbladder cancer [52]. AngII (angiotensin II): binds AT1R (pro-tumor: proliferation/angiogenesis [42]) or AT2R (context-dependent [45,54]). Ang(1-7): binds MasR (anti-tumor: inhibits metastasis [51]). AT1R (AngII type 1 receptor): pro-tumor; high expression correlates with poor colorectal cancer survival [44]. AT2R (AngII type 2 receptor): anti-tumor in non-inflamed tissues; pro-tumor under H. pylori infection [45,54]. MasR (Mas receptor): mediates Ang(1-7)’s anti-angiogenic effects [50]. Symbol meanings: → (activation); ⊣ (inhibition); orange (pro-tumor axis: ACE/AngII/AT1R); green (anti-tumor axis: ACE2/Ang(1-7)/MasR/AT2R); * organize/microenvironment specificity, may be because of inflammation in cancer of the stomach to promote tumor.

The established link between RAS and cancer progression is well documented [41]. Various retrospective analyses have highlighted the significant impact of RAS component dysregulation on numerous malignant tumors and its association with patient outcomes. For instance, elevated levels of both AT1R and AngII are frequently observed in aggressive ovarian adenocarcinomas, with increased AT1R expression closely linked to tumor malignancy [42]. Moreover, AngII levels correlate positively with the progression of surgical staging [43]. A study examining clinical and pathological data from 461 individuals with stage I–III colorectal cancer indicated that high AT1R expression correlates with a reduced rate of relapse-free survival [44]. RAS is also pivotal in various gastric cancer types, where ACE and AT2R show higher expression rates in primary tumors and lymph node metastases compared to healthy tissues [45]. Additionally, a comprehensive analysis of 31 gene expression studies, encompassing nearly 3200 microarray experiments, found that AT1R is overexpressed in 10% to 20% of breast cancer cases, a finding confirmed by multiple independent patient groups [46]. The intricate relationship between RAS element expression in tumors and cancer progression has been thoroughly explored. The ACE/AngII/AT1R pathway is elevated across a wide spectrum of malignancies, linking it to tumor growth and dissemination [47,48,49]. Conversely, the AngII/AT2R and angiotensin-converting enzyme 2 (ACE2)/angiotensin 1-7 (Ang(1-7))/Mas receptor (MasR) axis are negatively correlated with tumor proliferation and lymph node metastasis [50,51,52]. The tumor-suppressive function of AT2R is based on its ability to inhibit the ERK/PI3K pathway upon activation, thereby reducing cell proliferation and promoting apoptosis; its expression has been positively correlated with patient survival rates in both ovarian and lung cancers [51,53]. However, AT2R exhibits particularity in gastric cancer: while it is highly expressed in gastric cancer tissues, its effect is regulated by the inflammatory microenvironment. Specifically, in Helicobacter pylori infection-associated gastric cancer, lipopolysaccharide (LPS) activates the NF-κB pathway, leading to the binding of AT2R to β-arrestin2 and subsequent promotion of epithelial–mesenchymal transition (EMT). In non-infectious gastric cancer, AT2R still maintains its tumor-suppressive effect [45,53]. The relationship between dysregulation of RAS components and cancer development is summarized in Figure 1. Nonetheless, dysregulation of RAS components is not exclusive to cancer and may also manifest in other pathological states, indicating that it should not be solely viewed as a marker for cancer development. It remains unclear whether RAS dysregulation initiates malignant tumors or is a consequence of tumor evolution.

### 2.2. Progress in Targeted RAS Therapy for Cancer

Considerable advancements have been achieved in the application of RASi for cancer therapy. A Phase I clinical trial targeting RAS for aggressive tumors utilized Ang(1-7) as the primary anti-angiogenic agent; Ang(1-7) exerts this effect by specifically activating MasR, thereby inhibiting tumor angiogenesis [50]. This treatment was administered to patients with advanced solid tumors that are typically resistant to conventional therapies. The initial findings from this trial are promising; out of eighteen participants, one exhibited a 19% decrease in tumor size, while three others experienced clinical benefits with disease stabilization for over six months. Moreover, a comprehensive study involving 23 million individuals revealed that prolonged use of RASi, including ACE inhibitors (ACEis) and angiotensin receptor blockers (ARBs), was significantly associated with a lower risk of cervical and ovarian cancers, with adjusted odds ratios of 0.81 and 0.79, respectively [54]. Additionally, various retrospective analyses indicated that colorectal cancer patients receiving ACEis and/or ARBs had enhanced progression-free survival (PFS) and overall prognosis [44,55,56]. The efficacy of RASi as an adjunctive cancer treatment has been corroborated by numerous studies, which collectively analyzed 11,739 patients and reported a combined hazard ratio of 0.85 and a PFS of 0.91, highlighting the beneficial impact of RAS inhibitors on cancer patients [57]. This effect was notably stronger in individuals with urothelial and renal cell carcinomas, with hazard ratios of 0.53 and 0.56, respectively. However, despite extensive research, the application of RAS in oncology remains debated. Some studies argue that long-term use of ACEis/ARBs does not decrease cancer incidence [58], while others suggest that prolonged use of ARBs may marginally elevate the risk of specific cancers [54]. This inconsistency may arise from the expression patterns of RAS components in tumors and the types of RASi administered. For instance, in colorectal cancer patients, ACEis/ARBs therapy significantly enhanced PFS, particularly in those with left-sided colorectal cancer, due to the higher expression of AT1R in the left colon compared to the right [59]. The RAS comprises various axes and components, including ACE/AngII/AT1R, AngII/AT2R, and ACE2/Ang(1-7)/MasR, which interact with one another. For example, AngI can be cleaved into either AngII or Ang(1-7), with AngII typically produced by ACE, while Ang(1-7) can arise from AngII via ACE2 or from AngI through Ang(1-9), involving both ACE and ACE2 pathways [60]. Conversely, inhibiting RAS components such as ACE may lead to the accumulation of potentially detrimental substances like bradykinin or substance P [61]. Additionally, the role of AT2R, which is also present in tumors, complicates the scenario. Although AT2R is generally considered to exert anti-proliferative effects, AngII can, in certain contexts, stimulate cell proliferation through AT2R [53]. Therefore, understanding the expression profiles of RAS components in tumor tissues is crucial when employing RAS inhibitors for cancer treatment. Recent studies have demonstrated that using near-infrared fluorescent dyes, MPA, and radionuclide technetium-99m to label AngII achieves remarkable tumor-targeting specificity in the HepG2 mouse xenograft model, effectively identifying AT1R-positive tumors [62]. This discovery suggests that tumor molecular subtyping based on RAS component expression could represent a significant breakthrough for achieving precise RAS-targeted intervention.

### 2.3. Influence of the RASi in Cancer Biology

The improper regulation of the local RAS has been extensively recognized as a fundamental factor contributing to the aggressive progression of multiple cancers. It accelerates tumor growth through critical pathological mechanisms, including enhanced metastasis, cell adhesion, invasion, angiogenesis, proliferation, and epithelial–mesenchymal transition [63]. Observations from clinical settings and a wealth of data from animal studies consistently demonstrate that the primary effector molecule of RAS in cancer—particularly AngII—can directly promote tumor growth and dissemination by significantly stimulating angiogenesis [64,65]. Importantly, the increased metastatic and invasive potential of tumors associated with RAS dysregulation is likely supported by the abnormal angiogenesis resulting from this crucial pathological process. Additionally, various modulators targeting the RAS pathway, such as ACEis, ARBs, ACE2 agonists, AT2R agonists, and MasR agonists, have been shown to effectively induce apoptosis in different cancer cell types [66]. These findings indicate that components of the RAS pathway play a significant role in regulating either the apoptosis or proliferation of cancer cells.

## 3. Natural Polyphenols and Their Derivatives on RAS Regulation

### 3.1. Classification and Structural Characteristics of Polyphenols and Their Derivatives

**Figure 2:**
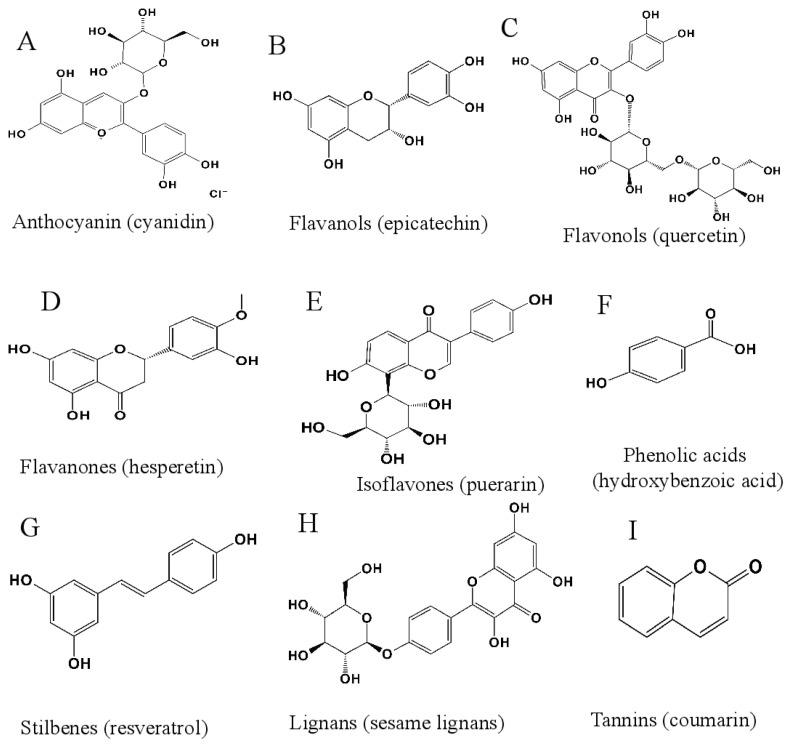
Structural characteristics of flavonoid and non-flavonoid polyphenols. (**A**–**E**) Flavonoid polyphenols (C6-C3-C6 benzopyran ring framework). (**A**) Cyanidin (anthocyanin): contains multiple hydroxyl groups (-OH) on the B-ring, which enhance its affinity for ACE and AT1R [78]. (**B**) Epicatechin (flavanol): the 3′-OH group on the B-ring contributes to its inhibitory effect on renin activity [40]. (**C**) Quercetin (flavonol): the C3-OH and C5-OH groups are critical for modifying thiol-containing transmembrane proteins (e.g., A disintegrin and metalloproteinase 17, (ADAM17)) [79]. (**D**) Hesperetin (flavanone): lacks a C2-C3 double bond, improving its metabolic stability in vivo [68]. (**E**) Puerarin (isoflavone): the isoflavone skeleton structure enables it to suppress renal AGT expression via antioxidant activity [80]. (**F**–**I**) Non-flavonoid polyphenols. (**F**) Hydroxybenzoic acid (phenolic acid): the carboxyl group (-COOH) can form hydrogen bonds with the active site of ACE, enhancing inhibitory activity [81]. (**G**) Resveratrol (stilbene): the trans-stilbene structure is essential for upregulating ACE2 expression via the Sirtuin 1 pathway [82]. (**H**) Sesame lignans (lignan): the diphenylpropane skeleton enhances its bioavailability and ability to regulate gut microbiota-mediated polyphenol metabolism [38]. (**I**) Coumarin (tannin): the lactone ring structure contributes to its inhibition of AT1R expression [68].

Polyphenols represent a diverse group of organic compounds found throughout the plant kingdom, characterized by the presence of aromatic rings and a minimum of two phenolic hydroxyl groups. The fundamental building blocks of polyphenols are phenolic units, which can be connected in various configurations to create different isomers or derivatives. Based on their chemical structure and origin, natural polyphenols can be categorized into two main groups: flavonoids and non-flavonoids. Flavonoids, the most prevalent and extensively researched phenolic compounds in plants, feature a C6-C3-C6 framework, also known as the benzopyran ring structure. Key types of flavonoids include anthocyanins (e.g., cyanidin), flavanols (e.g., epicatechin), flavonols (e.g., quercetin), flavanones (e.g., hesperetin), and isoflavones (e.g., puerarin) [67,68,69,70]. Non-flavonoids are phenolic compounds that do not conform to the flavonoid skeleton and encompass a variety of structural types, including (1) phenolic acids, such as hydroxybenzoic and hydroxycinnamic acids along with their derivatives [68,71,72]; (2) stilbenes/stilbenoids, including resveratrol [69,70,73]; (3) lignans, some of which possess a Flavanolignan structure, like sesame lignans; and (4) tannins, consisting of galloylated catechins, xanthones, and coumarins [68,70]. The classification of polyphenol derivatives primarily relies on the method of modification, which includes acyl derivatives, microbial metabolic derivatives, and other structurally altered derivatives. Acyl derivatives are formed through the enzymatic esterification of a water-soluble polyphenol, such as EGCG, resulting in a lipophilic derivative by losing a hydroxyl or carboxyl group [74,75]. Microbial metabolic derivatives are produced by intestinal flora that convert polyphenols into more biologically active forms [38]. Other structurally modified derivatives encompass glycosides and quinones [76,77]. Figure 2 illustrates distinctive structures of flavonoids and non-flavonoids. This section emphasizes the regulatory roles of polyphenols and their derivatives on critical components of the RAS and the biaxial balance.

### 3.2. Natural Polyphenols and Their Derivatives Regulate RAS Biaxial Balance

Natural polyphenols and their derivatives exhibit considerable promise in modulating the balance of RAS dual-axis. Isoflavones possess the ability to suppress local angiotensin (AGT) expression in the kidneys through their antioxidant properties, potentially slowing the progression of diabetes [80]. Flavonoid polyphenols, in particular, can diminish the expression of AT1R, thereby counteracting the synthesis of AngII. Compounds such as rosmarinic acid [83], along with polyphenolic derivatives like myricetin and kaempferol, demonstrate a strong affinity for AT1R [84] and function as ARBs. These flavonoids can reduce AngII production by inhibiting ACE, and even natural polyphenols have been shown to influence ACE activity in a structure-dependent manner [78,81]. The overproduction of AngII activates NADPH oxidase (NOX), resulting in an increase in reactive ROS and a decrease in endothelial nitric oxide (NO). Blueberry polyphenol extract (BPE) has been identified as a counteractive agent against the reduction in NO levels induced by AngII, thereby enhancing endothelial function [85]. Furthermore, studies indicate that resveratrol can inhibit the AngII signaling pathway by lowering serum AngII levels and reducing the expression of ACE and the renin receptor. Concurrently, it promotes the expression of ACE2 and the AngII type 2 receptor (AT2R), mitigating the detrimental effects of AngII [86]. In experimental models of abdominal aortic aneurysm, resveratrol significantly elevates ACE2 levels in both serum and tissues, activating ACE2 via the Sirtuin 1-dependent pathway, which aids in inhibiting aneurysm development [82]. Glycyrrhetinic acid, a triterpene derivative, interacts with the P-loop domain of RAS, modifying its stability and preventing excessive activation of the classical axis (ACE/AngII/AT1R axis) by decreasing renin and ACE activity [87,88]. Notably, intestinal microbiota convert polyphenols into microbial metabolites such as phenylpropionic acid derivatives, enhancing their biological activity and absorption, which further influences RAS [38,89]. In summary, polyphenols achieve a dynamic balance of the RAS dual-axis by inhibiting the excessive activation of the classical axis (ACE/AngII/AT1R axis), through the reduction in renin, ACE, and AT1R activity, while simultaneously enhancing the protective axis (ACE2/Ang(1-7)/MasR axis) by upregulating ACE2, Ang(1-7), and MasR, thereby alleviating hypertension, tumors, cardiovascular diseases, and other pathological conditions.

### 3.3. Natural Polyphenol Oxidative Products Regulate RAS

Beyond polyphenols and their derivatives, the oxidation products of polyphenols have been shown to influence the regulation of RAS. These compounds, characterized by phenolic hydroxyl groups, readily undergo oxidation and polymerization, leading to the formation of polymers. Research conducted by Wu et al. indicated that the autoxidation products of EGCG, referred to as EAOP, exhibit a more pronounced effect on RAS regulation compared to their unoxidized form, as they are generated through autoxidation under alkaline conditions (pH 8.0 phosphate buffer) [90]. EAOP contributes to the maintenance of RAS balance, resulting in therapeutic outcomes in type 2 diabetic mice by inhibiting the pro-diabetic pathway—specifically, by reducing levels of ACE, AngII, AT1R, and renin in the kidney and liver—while enhancing the protective pathway through the elevation of ACE2, AT2R, and MasR, particularly in the liver, adipose tissue, and pancreatic islets [90]. Additionally, Yang et al. discovered that EAOP produced via the same oxidation method decreased cell viability in a dose-dependent manner (IC_50_: 800 μg/mL for CaCo2, 300 μg/mL for TCA8113). EAOP induced cell apoptosis by suppressing the pro-tumor axis (ACE/AngII/AT1R), activating the anti-tumor axis (ACE2/Ang(1-7)/MasR), and elevating AGT levels [59]. The oxidative polymerization of polyphenols and their derivatives may confer health benefits, including hypoglycemic and anti-cancer effects, through precise bidirectional regulation of RAS.

### 3.4. Anti-Tumor Effects of Polyphenols by Regulating RAS

**Figure 3:**
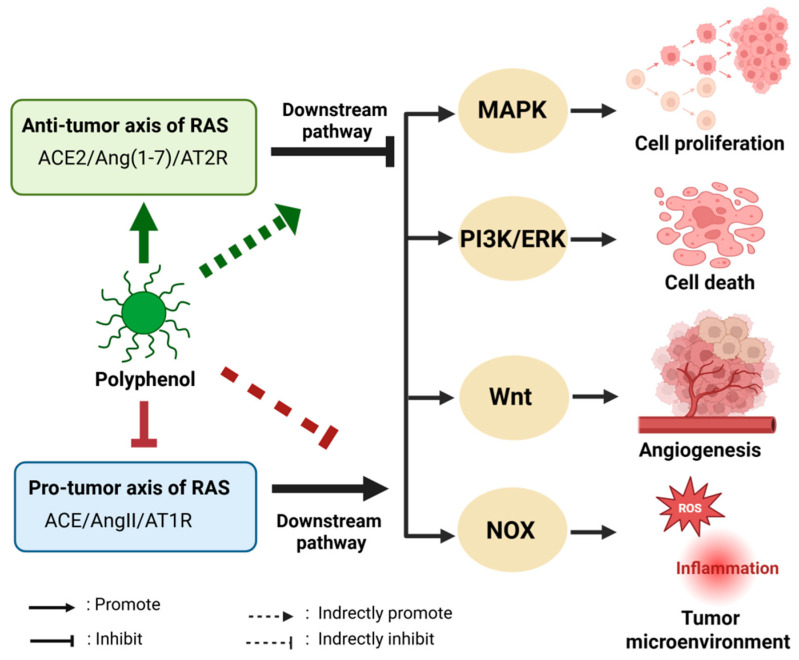
Schematic of polyphenols inhibiting pro-tumor signaling cascades by regulating RAS dual-axis balance. Polyphenols: the central regulatory molecule (e.g., EGCG, resveratrol), which inhibits the pro-tumor axis and activates the anti-tumor axis [58,90]. RAS dual-axis: pro-tumor axis (ACE/AngII/AT1R), activates downstream pathways including NOX (inducing oxidative stress and inflammation) and Wnt (promoting angiogenesis and metastasis) [95,97]. Anti-tumor axis (ACE2/Ang(1-7)/AT2R): inhibits downstream pro-tumor pathways such as MAPK (reducing cell proliferation) and PI3K/ERK (inducing cell death) [51,99]. Tumor microenvironment indicates the comprehensive regulatory effect of polyphenols on the tumor microenvironment (e.g., reducing pro-inflammatory cytokines) [39].

Polyphenols can interfere with specific aspects of the RAS pathway to exert anti-tumor effects. Firstly, polyphenols can reduce the expression of AT1R, which diminishes the activation of MAPK mediated by AT1R, resulting in decreased cell growth and the promotion of apoptosis. The MAPK pathway, a crucial signaling route downstream of RAS, includes key molecules such as Ras, RAF, MEK, and ERK, which are essential for regulating cell growth, survival, and differentiation. In cancer, excessive RAS activation, often through AT1R or (P)RR, can lead to abnormal MAPK activation, causing uncontrolled tumor cell growth [91]. Certain polyphenols, including specific flavonoids, can directly influence MAPK signaling by inhibiting ERK phosphorylation, which in turn reduces tumor cell proliferation. For example, in models of colorectal cancer (CRC), polyphenols affect pathways such as MAPK/PI3K, effectively blocking pro-tumor signals [92]. Additionally, compounds like resveratrol have been found to indirectly disrupt the RAS-MAPK pathway by inhibiting upstream EGFR, which is linked to MAPK, thereby slowing tumor progression [93]. Secondly, polyphenols can decrease AngII levels, which indirectly suppresses PI3K activation, leading to reduced cell growth and increased apoptosis. The PI3K/AKT/mTOR pathway is vital for regulating cell growth, autophagy, and resistance to apoptosis, acting as a significant downstream effector of RAS. RAS activation through AngII can enhance PI3K and mTOR activity, supporting tumor survival and metabolic adaptation [91]. For instance, chronic RAS signaling can affect cell growth via mTOR, which is associated with the anti-apoptotic properties of tumor cells [94]. Polyphenols such as curcumin and resveratrol can target the PI3K/AKT/mTOR pathway, inhibiting AKT phosphorylation and mTOR function, thereby promoting apoptosis in tumor cells. This has been clearly demonstrated in CRC, where polyphenols counteract the proliferation and anti-apoptotic characteristics of tumor cells by modulating PI3K signaling [92,95]. This action may be linked to the inhibition of RAS input, as downstream signaling from AT1R can interact with the PI3K pathway. Furthermore, polyphenols have been shown to reduce AT1R and Wnt signaling, thereby inhibiting processes such as angiogenesis and metastasis [95,96]. The Wnt/β-catenin pathway plays a crucial role in cell differentiation and gene expression during tumor development, demonstrating a close relationship with RAS, which collectively facilitates tumor microenvironment fibrosis and progression [92,97]. For instance, Wnt signaling can enhance downstream RAS components, contributing to renal fibrosis and cancer-associated chronic kidney disease, as evidenced in a 5/6 nephrectomy model [97]. Polyphenols, including curcumin, can inhibit the Wnt/β-catenin pathway, disrupting its downstream effects, such as the nuclear translocation of β-catenin, thereby reducing tumor growth and metastasis. In CRC, the modulation of Wnt signaling by polyphenols has been confirmed as part of their anti-cancer mechanism, which includes the suppression of RAS-related gene overexpression [92]. Additionally, polyphenols can mitigate oxidative stress and inflammation by reducing AT1R expression or activity, which subsequently inhibits RAS-associated NOX and lowers ROS levels [39]. In tumors, the activation of AT1R increases ROS through NOX, which can impair endothelial function and stimulate cell proliferation [39]. Polyphenols can indirectly inhibit this downstream pathway by decreasing AT1R expression or activity. For example, polyphenols have been shown to reduce oxidative stress and inflammation through AT1R downregulation in hypertension models [98]; this mechanism may also apply to the tumor microenvironment, decreasing ROS-induced DNA damage and angiogenesis. Compounds such as resveratrol have been demonstrated to target various signaling pathways, including those associated with AT1R, thereby inhibiting tumor growth [95]. Moreover, regarding anti-tumor activity, the ACE2/Ang-(1-7)/MasR axis collaborates with AT2R to establish a protective axis that inhibits tumor growth, angiogenesis, and metastasis by downregulating pro-tumor pathways, such as ERK and PI3K/Akt, associated with the classical RAS axis (ACE/AngII/AT1R). This axis also protects the tumor microenvironment by reducing pro-inflammatory cytokine levels and alleviating oxidative stress [51,99,100,101,102]. Polyphenols may indirectly influence Ang(1-7) signaling by modulating the balance of RAS. For instance, the anti-inflammatory effects of polyphenols could enhance MasR activity, thereby augmenting anti-tumor effects [39,96]. In conclusion, polyphenols exert their anti-cancer effects by regulating the dual-axis balance of RAS, effectively inhibiting pro-tumor signaling pathways, including the downstream MAPK, PI3K/mTOR, and Wnt/β-catenin pathways (Figure 3).

## 4. Mechanisms of Natural Polyphenols in Regulating RAS

### 4.1. Polyphenols Regulate RAS by Modifying Thiol-Containing Transmembrane Proteins on the Cell Membrane

Recent studies indicate that the regulation of RAS components may be influenced by specific extracellular transmembrane proteins [103,104,105]. These proteins contain unique thiol switches within their ectodomains, which facilitate rapid intracellular signaling in response to changes in the surrounding environment [106]. The activity and structure of a protein from the disintegrin and metalloprotease domain family, known as ADAM17, can be modulated by a thiol switch located in its ectodomain [106]. Notably, a connection between ADAM17 and RAS has been identified under high glucose conditions [103]. Specifically, exposure to high glucose levels resulted in increased expression of ADAM17 and AT1R in primary cardiofibroblasts. When ADAM17 was knocked down in these cells, ACE2 levels increased while AT1R expression decreased. Similarly, in the hearts of diabetic mice, both ADAM17 and AT1R levels were elevated, and knocking down ADAM17 led to increased ACE2 and decreased AT1R expression. In another investigation, overexpression of dickkopf-3 (DKK3) significantly enhanced ACE2 levels and promoted the degradation of AngII by inhibiting the phosphorylation of ADAM17, thereby reducing cardiac hypertrophy and fibrosis induced by AngII infusion in a mouse model with adenovirus-mediated DKK3 overexpression [104]. Additionally, integrin α2β1, another transmembrane protein with thiol groups, has been implicated in promoting cancer cell growth, proliferation, and invasion through its regulation of collagen 1. In Caki-1 renal cancer cells treated with the integrin α2β1 inhibitor BTT3033, a significant decrease in the activation of integrin α2β1 was observed [105]. Furthermore, inhibiting integrin α2β1 resulted in a marked reduction in the expression of ACE and AT1R as well as decreased phosphorylation of the pro-proliferative signaling pathways AKT and ERK 1/2. In mouse models, the targeted deletion of integrin β1 in renal proximal tubules also led to a significant decrease in ACE2 expression within kidney tissues [107].

**Figure 4:**
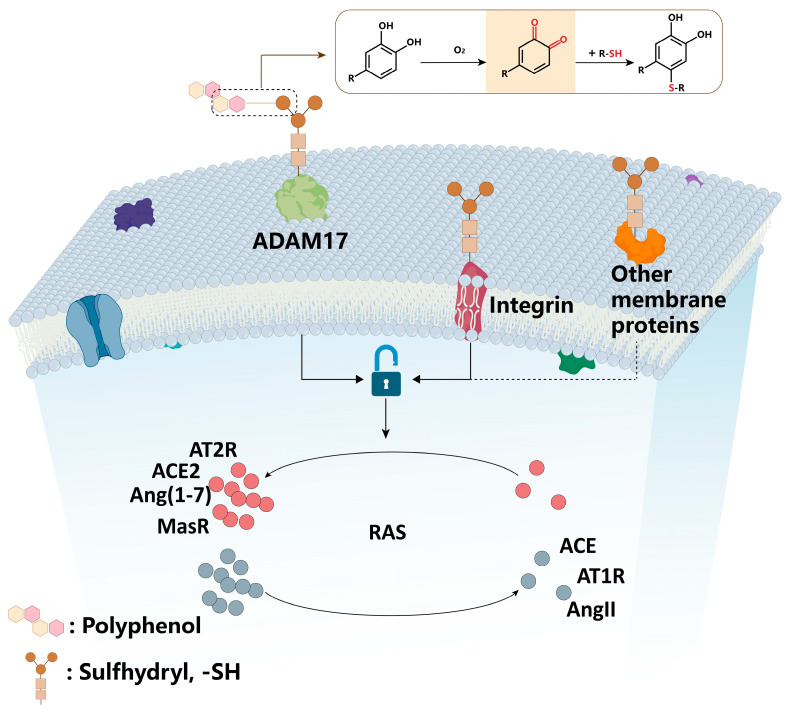
Schematic of polyphenols regulating RAS by modifying thiol-containing transmembrane proteins. Polyphenols rapidly bind to cell surfaces and accumulate within 120 s [58,90]. Thiol-containing transmembrane proteins: (1) ADAM17: its phosphorylation is inhibited by polyphenols (e.g., rosmarinic acid), reducing ACE2 shedding and AT1R expression [79,103]. (2) Integrins (e.g., α2β1): polyphenols (e.g., carnosic acid) reduce their expression, decreasing ACE and AT1R levels and inhibiting AKT/ERK pathway activation [105,110]. RAS components: ACE, AT1R (pro-tumor), ACE2, AT2R, Ang(1-7), and MasR (anti-tumor), whose expression or activity is regulated by modified transmembrane proteins [107]. The dotted lines in the figure indicate that polyphenols may also bind to other membrane proteins containing sulfhydryl groups and thereby regulate the expression of RAS.

Research has demonstrated that polyphenols and their oxidized derivatives interact with thiol groups. For instance, EGCG and its oxidation products have been shown to prevent lysozyme fibrillation [108], a process involving the modification of thiols by quinones or similar compounds generated from EGCG [109]. Laboratory studies further established that EGCG and its oxidized forms can specifically modify thiol-containing substances, such as DTT, Cys, and GSH, as well as proteins with sulfhydryl groups, including ribonuclease [59]. These findings lead us to hypothesize that EGCG and its oxidation products may react with sulfhydryl groups located in the ectodomains of transmembrane proteins, potentially including ADAM17 and integrin α2β1, thereby influencing their structure and function, which could subtly modulate the RAS axis. Supporting this hypothesis, polyphenols and their oxidation products exhibit a strong tendency to bind to cell or tissue surfaces, quickly adhering rapidly within 10 s and accumulating significantly within 120 s [59,90]. Additionally, studies indicate that rosmarinic acid can inhibit the growth and movement of melanoma cells, promote apoptosis, and increase sensitivity to cisplatin by targeting the ADAM17/EGFR/AKT/GSK3β pathway [79]. Notably, the overexpression of ADAM17 counteracted the effects of rosmarinic acid, while the use of the ADAM17 inhibitor TACE produced opposite results. Another polyphenol, carnosic acid, has been found to reduce integrin expression, thereby hindering α4 and α9 integrin-dependent cell adhesion and effectively blocking melanoma metastasis in a mouse lung model [110]. Schematic of polyphenols regulating RAS by modifying thiol-containing transmembrane proteins is presented in Figure 4. Although current evidence strongly indicates that polyphenolic compounds can influence transmembrane proteins like ADAM17 and integrins through thiol interactions, more comprehensive studies are required to elucidate how these polyphenol–thiol interactions regulate components of the RAS pathway.

### 4.2. Direct Binding of Polyphenols to the Active Site of RAS Components

Natural polyphenols have been demonstrated to inhibit ACE, a pivotal enzyme in the detrimental RAS axis. A distinct correlation exists between the structural characteristics of these polyphenols and their ACE inhibitory capacity [78,81]. Typically, hydrophobic benzene rings enhance the inhibitory effect, as observed in compounds such as hydroxybenzoic and hydroxycinnamic acids, whereas hydroxymethylation often reduces activity due to steric hindrance. The presence of carboxylic or acrylic acid groups, which can accept hydrogen bonds, further augments inhibition. Flavonoid glycosides containing sugar groups in the C-ring generally exhibit superior inhibitory activity compared to their aglycone counterparts. Notably, low IC_50_ values for certain flavonoids, including 3,7-dihydroxyflavone (0.04 µmol/L), fisetin (0.08 µmol/L), acteoside (0.36 µmol/L), and anthocyanins such as delphinidin-3-O-sambubioside (0.14 µmol/L) and cyanidin-3-O-sambubioside (0.11 µmol/L), indicate a direct interaction with the catalytic zinc ion, effectively obstructing substrate access and hydrolysis [78,111,112]. Compounds like rosmarinic acid (0.05 µmol/L) and phenolic acid derivatives likely compete for the active site by mimicking natural peptide substrates of ACE, including angiotensin I and bradykinin [78,81]. The structural diversity of effective inhibitors suggests that they exploit various interactions within the active site pocket. Molecular docking simulations frequently reveal multiple hydrogen bonds between potent polyphenolic inhibitors (such as geraniin (IC_50_ = 13.22 µM), eupatorin (15.35 µg/mL), and sinensetin (29.5 µg/mL)) and ACE residues. Glycosylated flavonoids, such as rutin (0.45 µmol/L), may utilize sugar moieties for additional hydrogen bonding [81,111,112]. Docking analyses emphasize hydrophobic interactions between inhibitors like polymethoxyflavones (eupatorin, sinensetin, 3′-hydroxy-5,6,7,4′-tetramethoxyflavone) and hydrophobic residues (such as Phe, Trp, Val) in the S1 and S2 subsites of ACE. Methylation of flavonoids can further enhance these interactions and their potency [111,112]. This subsection systematically organizes the inhibitory concentrations and effects of natural polyphenol complexes, extracts, and derivatives on ACE, as detailed in Table 1.

The influence of polyphenols on components of RAS has been well established, particularly concerning the AT1R receptor. Rosmarinic acid, a polyphenolic compound found in rosemary, directly interacts with the AT1R receptor by binding to critical sites such as His6 and Pro7, leading to effects that counteract those of synthetic ARBs [83]. Research aimed at identifying natural ARB inhibitors has demonstrated that polyphenolic compounds, including myricetin and kaempferol, exhibit a strong affinity for AT1R [84]. Computational analyses suggest that these compounds bind more effectively than the conventional AT1R inhibitor olmesartan [84]. Furthermore, experimental findings indicate that quercetin can significantly mitigate the physiological effects induced by AngII by inhibiting the activation pathway of AT1R [116]. Additionally, high-polyphenol fractions derived from mulberry, particularly the ethyl acetate fraction, have been shown to reduce oxidative stress by enhancing overall antioxidant capacity, which subsequently influences the expression and activity of detrimental RAS components, such as renin and AT1R, in mice subjected to a high-salt diet [117].

Research has revealed the presence of ACE, ACE2, AT1R, and AT2R on the surface of cell membranes [41]. This finding not only supports the possibility that polyphenols may regulate their activity through direct binding to membrane-anchored RAS components (e.g., ACE and AT1R) but also provides new insights into the molecular mechanisms underlying polyphenol-mediated RAS modulation. Nonetheless, this proposed mechanism requires further exploration using methodologies such as molecular docking and surface plasmon resonance. Furthermore, the potential interactions between polyphenols and other membrane-associated RAS components (such as ACE2 and AT2R) warrant thorough examination.

## 5. Clinical Research

### 5.1. Introduction to RAS-Targeted Clinical Evidence

The dysregulation of RAS is a well-established driver of cancer progression. Preclinical studies consistently demonstrate that the imbalance between the pro-tumor ACE/AngII/AT1R axis and the anti-tumor ACE2/Ang(1-7)/MasR axis promotes angiogenesis, cell proliferation, and metastasis [41,44]. Components of RAS, such as AT1R and ACE, are abnormally expressed in various malignancies, ranging from CC-RCC to triple-negative breast cancer (TNBC), and their expression correlates with poor patient outcomes. This clinical relevance has spurred research into RAS inhibitors, including ACEi and ARBs, as potential anti-cancer agents, yielding preliminary evidence of survival benefits across different cancer types. However, a deeper exploration is needed to link the clinical findings of RAS inhibitors to the role of natural polyphenols as RAS modulators in cancer therapy. Below, we focus on analyzing key clinical and preclinical studies that validate RAS targeting in cancer and bridge these insights to the preclinical mechanisms of polyphenol-RAS regulation.

### 5.2. Cancer-Specific Clinical/Preclinical Analyses of RAS Targeting

Yoshida et al. conducted a retrospective cohort study involving 823 patients diagnosed with muscle-invasive bladder cancer who underwent radical cystectomy, a procedure associated with a high risk of post-surgical recurrence. The study stratified patients into two groups: those who used RASi, specifically ACEi or ARBs, for comorbid conditions such as hypertension and non-users. With a 5-year follow-up period, the primary endpoints were cancer-specific mortality (CSM) and overall mortality (OM). The results indicated that RASi use was linked to a 53% reduction in CSM (hazard ratio [HR] = 0.47, 95% confidence interval [CI] 0.32–0.68) and a 64% reduction in OM (HR = 0.36, 95% CI 0.25–0.52). Subgroup analysis revealed that ARBs, such as losartan, provided greater survival benefits than ACEi (CSM HR: 0.42 vs. 0.85), which is consistent with findings in Section 3.2 that highlight the more specific inhibition of pro-tumor AT1R by ARBs [44]. Mechanistically, analyses of tumor tissue and serum samples indicated that RASi reduced AT1R expression in residual bladder cancer cells, thereby suppressing vascular endothelial growth factor (VEGF) secretion [118]. This finding aligns with Section 3.4, which posits that AT1R activation drives VEGF-dependent angiogenesis, a process also inhibited by polyphenols, such as rosmarinic acid, which binds to AT1R to block downstream VEGF signaling [83]. These results validate AT1R as a therapeutic target for bladder cancer and suggest that polyphenols could complement RASi therapy to enhance AT1R inhibition without additional toxicity.

Dougherty et al. combined preclinical and early clinical data to investigate the efficacy of RASi, specifically losartan (an ARB), in conjunction with vitamin D for the treatment of CRC, a prevalent malignancy characterized by well-documented RAS dysregulation [6,44]. The study consisted of two components: in the preclinical phase, Apc gene-knockout mice, a model for sporadic CRC, were treated with losartan alone, vitamin D alone, losartan + vitamin D, or vehicle; in the clinical phase, 40 patients with early-stage CRC were administered either the losartan–vitamin D combination or standard surveillance. Key findings indicated that, preclinically, the combination treatment resulted in a 42% reduction in the number of intestinal tumors compared to controls, with tumor lysis adverse event rates demonstrating downregulation of AT1R (the pro-tumor axis) and upregulation of ACE2 (the anti-tumor axis), confirming dual RAS modulation. Clinically, patients receiving the combination therapy exhibited a median PFS of 11.2 months, which is 3.6 months longer than that of the control group (7.6 months), without an increase in adverse events (AEs) such as hypotension or hypercalcemia. Mechanistically, the observed synergy between losartan and vitamin D is attributed to complementary regulation of RAS: losartan blocked AT1R to suppress the pro-tumor axis, while vitamin D enhances ACE2 levels, thereby promoting the anti-tumor ACE2/Ang(1-7)/MasR axis [119]. This finding parallels the results in Section 3.2, which demonstrate that polyphenols, such as resveratrol, also upregulate ACE2 through Sirtuin 1-dependent pathways [82]. This supports the potential of ‘RAS-modulating combinations’ in CRC treatment and suggests that polyphenols (e.g., EGCG, resveratrol) could either replace or augment vitamin D in such therapeutic regimens due to their shared capacity to upregulate ACE2.

Vallejo Ardila et al. conducted a systematic review and meta-analysis of 11 observational studies involving 3200 patients with non-small cell lung cancer (NSCLC), the most prevalent subtype of lung cancer often characterized by EGFR mutations. Their study evaluated the association between the use of RASi, specifically ACEi and ARBs, and survival outcomes, including overall survival (OS) and progression-free survival (PFS), with subgroup analyses stratified by EGFR mutation status. The key findings indicated that, overall, RASi use was associated with a 17% reduction in the risk of OS (HR = 0.83, 95% CI 0.75–0.92) and a 9% improvement in PFS (HR = 0.91, 95% CI 0.84–0.99). Notably, the survival benefit was more pronounced in the EGFR-mutant subgroup, with an OS HR of 0.71 (95% CI 0.62–0.81), suggesting a potential synergy between RASi and EGFR-targeted therapies. The meta-analysis attributed the efficacy of RASi to two RAS-dependent mechanisms: (1) tumor vascular normalization, where RASi inhibited AT1R-mediated abnormal angiogenesis to enhance drug delivery, and (2) immune modulation, whereby RASi increased CD8+ T cell infiltration, potentially enhancing the response to immune checkpoint inhibitors [120]. These findings align with preclinical data, as noted in Section 3.4, which indicates that polyphenols (e.g., EGCG, curcumin) reduce tumor angiogenesis through AT1R inhibition, while Section 4.1 highlights their ability to modulate the tumor microenvironment by decreasing oxidative stress and inflammation [38,39]. Thus, the study confirms RAS as a valid therapeutic target for NSCLC and positions polyphenols as promising adjuvants to immunotherapy or EGFR inhibitors.

Renziehausen et al. investigated the role of RASi, specifically valsartan, an ARB, in BRAF-mutant melanoma—a subtype characterized by a high metastatic potential and frequent resistance to BRAF inhibitors such as vemurafenib. The study encompassed both in vitro and in vivo experiments, utilizing BRAF-mutant melanoma cell lines A375 and SK-MEL-28 treated with valsartan alone or in combination with vemurafenib, as well as immunocompromised mice xenografted with A375 cells receiving the same treatment regimens. Key findings revealed that valsartan reduced AT1R expression by 40% and inhibited cell invasion by 52% in vitro, as demonstrated by transwell assays, compared to the vehicle control. In vivo, the combination of valsartan and vemurafenib achieved an objective response rate (ORR) of 68% compared to 45% for vemurafenib alone, and it inhibited tumor growth by 62%. Mechanistically, AT1R activation in melanoma cells enhances the expression of matrix metalloproteinase-9 (MMP-9), a crucial mediator of invasion and metastasis, through the activation of the MAPK pathway [121].Valsartan inhibited this signaling cascade via AT1R blockade, thereby reversing resistance to BRAF inhibitors. This finding is consistent with Section 3.4 of the paper, which indicates that polyphenols, such as quercetin and curcumin, also inhibit MMP-9 and MAPK signaling through RAS modulation [25,116]. This suggests that polyphenols may serve as low-toxicity alternatives to RASi for enhancing the efficacy of targeted therapies in melanoma.

de Miranda et al. conducted a retrospective cohort study involving 120 patients diagnosed with TNBC, an aggressive subtype characterized by the absence of hormone receptors and HER2 targets, resulting in limited treatment options. The study stratified patients based on two factors: (1) AT1R expression levels (high/low, assessed through immunohistochemistry) and (2) the use of RAS inhibitors (specifically losartan), with disease-free survival (DFS) serving as the primary endpoint, as it is a surrogate for recurrence risk. Key findings indicated that in the AT1R-high subgroup, patients treated with losartan experienced a 35% reduction in recurrence risk (HR = 0.65, 95% CI 0.51–0.83) and an extension of 2.8 years in median DFS (6.5 years in users versus 3.7 years in non-users). In contrast, no significant survival benefit was observed in the AT1R-low subgroup, thereby affirming AT1R as a predictive biomarker for the efficacy of RAS inhibitors. Mechanistically, tumor molecular analysis showed that AT1R activation stabilizes β-catenin, a crucial mediator of the Wnt pathway that drives TNBC metastasis [122]. Furthermore, losartan was found to disrupt this interaction, leading to reduced nuclear translocation of β-catenin and decreased expression of downstream pro-metastatic genes. This finding directly supports Section 3.4, which illustrates that polyphenols (e.g., EGCG) also inhibit the Wnt/β-catenin pathway through RAS modulation [97], thereby identifying AT1R-high patients as optimal candidates for RAS-targeted therapies, including polyphenols that can concurrently inhibit both AT1R and Wnt signaling in TNBC.

### 5.3. Translational Implications for Polyphenols

Clinical and preclinical evidence supports the targeting of RAS as a viable anti-cancer strategy [118,119,120,121,122]. These insights suggest the potential of natural polyphenols as RAS-modulating agents. Below, we summarize the alignment between the clinical benefits of RAS inhibitors and the mechanisms of polyphenols, and we propose actionable clinical trial designs to advance polyphenol-based cancer therapies.

The five studies highlighted that RASi exert their efficacy through two core mechanisms: (1) the inhibition of the pro-tumor ACE/AngII/AT1R axis and (2) the enhancement of the anti-tumor ACE2/Ang(1-7)/MasR axis. These mechanisms are also shared by natural polyphenols, as documented in Section 3 and Section 4. For AT1R inhibition, polyphenols such as rosmarinic acid and quercetin bind to AT1R with high affinity, effectively blocking AngII-mediated pro-tumor signaling [83,116], which mirrors the effects of RASi (e.g., losartan) observed in bladder cancer and TNBC [118,122]. Regarding ACE2 upregulation, resveratrol and EGCG enhance ACE2 expression via Sirtuin 1 or antioxidant pathways [82,90], similar to the role of vitamin D in augmenting RASi efficacy in CRC [119]. Furthermore, in terms of microenvironment modulation, polyphenols reduce oxidative stress and inflammation by inhibiting NADPH oxidase (NOX) (Section 3.4), aligning with RASi’s capacity to normalize the tumor microenvironment in NSCLC [120]. Notably, polyphenols present several advantages over synthetic RASi: they exhibit low toxicity even at high doses [123], can be administered orally, and modulate multiple components of the RAS simultaneously (e.g., EGCG inhibits ACE and AT1R while upregulating ACE2 [59,90]).

Based on RASi clinical data, three Phase II trial designs are proposed to validate the efficacy of polyphenols in cancer treatment: (1) The first trial targets metastatic CRC, building on the synergy between losartan and vitamin D. This trial would enroll 150 patients stratified by AT1R expression. The experimental arm will receive EGCG (1000 mg/day, a well-tolerated dose [123]) in conjunction with losartan (50 mg/day), while the control arm will receive losartan alone. The primary endpoint will be progression-free survival (PFS), with changes in RAS component expression (ACE, ACE2, AT1R) in plasma and tumor biopsies serving as secondary endpoints. (2) The second trial focuses on adjuvant therapy for AT1R-high TNBC, identifying AT1R as a biomarker. This trial will enroll 120 patients with resected AT1R-high TNBC, where the experimental arm will take resveratrol (500 mg/day) for one year, and the control arm will receive standard surveillance. The primary endpoint will be DFS, with safety and changes in serum AngII levels as secondary endpoints. (3) The third trial is designed for advanced NSCLC, where RASi enhances immunotherapy. This trial will enroll 200 patients (either EGFR-mutant or wild-type), with the experimental arm receiving a polyphenol cocktail (EGCG + resveratrol + quercetin) combined with an anti-PD-1 antibody, while the control arm will receive anti-PD-1 alone. The primary endpoint will be the ORR with overall survival (OS) and CD8+ T cell infiltration (measured via immunohistochemistry) as secondary endpoints.

While the RASi clinical data lay a solid foundation for RAS-targeted cancer therapy, several limitations must be addressed to advance polyphenol-based interventions: (1) Early clinical studies have small sample sizes, which limits generalizability; therefore, future polyphenol trials should adopt larger, multi-center cohorts to validate efficacy. (2) There is a lack of polyphenol-specific clinical data, as all current evidence for RAS modulation in cancer derives from synthetic RAS inhibitors, with no phase II/III trials evaluating polyphenols as RAS-targeted agents. Prior to large-scale trials, phase I studies should define the optimal doses of polyphenols (e.g., EGCG, resveratrol) when combined with RAS inhibitors or immunotherapies and assess pharmacokinetics (e.g., bioavailability, tissue distribution [124]). (3) Biomarker-driven stratification is essential—References [120,122] highlight the importance of RAS component expression (e.g., AT1R, EGFR) in predicting treatment response. Therefore, future polyphenol trials should stratify patients by RAS subtype (e.g., ACE-high vs. AT1R-high) to ensure targeted delivery, which is supported by preclinical data demonstrating that polyphenol efficacy depends on structure and RAS subtype [78,90]. (4) There are challenges in polyphenol delivery—polyphenols exhibit low bioavailability and poor targeting, as discussed in Section 6. Future research should integrate nanodelivery systems (e.g., PLGA nanoparticles [125]) to enhance polyphenol accumulation in tumors, as preclinically demonstrated for EGCG [126]. Addressing these limitations is critical for translating preclinical polyphenol-RAS research into clinical practice. With rigorous trial design and biomarker integration, polyphenols have the potential to become low-toxicity, RAS-targeted adjuvants for various cancer types.

## 6. Challenges and Translational Opportunities for RAS-Targeted Cancer Therapy

The exploration of natural polyphenols as RAS-modulating agents for cancer therapy presents significant potential to address unmet clinical needs; however, it also encounters inherent challenges. We summarize key barriers to translation and then focus on patient-centric translational opportunities, each linked to tangible improvements in treatment efficacy, reduced toxicity, or enhanced quality of life for cancer patients.

### 6.1. Key Challenges to Translating RAS-Polyphenol Research

Preclinical data strongly support the role of polyphenols in targeting RAS pathway for cancer therapy. However, three core challenges hinder their clinical application. First, polyphenols (e.g., EGCG, resveratrol) exhibit low bioavailability and poor tumor targeting due to low water solubility and rapid in vivo metabolism, resulting in subtherapeutic concentrations at tumor sites. For instance, oral administration of EGCG yields a maximum plasma concentration (C_max_) of only 0.5–2 μmol/L, which is significantly below the IC_50_ values (0.04–0.45 μmol/L) required for RAS inhibition [78,123]. Second, there is a lack of clinical evidence specific to polyphenols. All current data on RAS-targeted cancer therapies stem from synthetic RAS inhibitors (ACEI/ARBs), and no phase II/III trials have evaluated polyphenols as standalone or adjuvant RAS modulators. This gap limits the validation of the efficacy of polyphenols in patient populations. Third, there is a need for biomarker-guided patient selection. Studies [120,122] indicate that the expression of RAS components (e.g., AT1R, EGFR) can predict treatment response; however, no standardized tools currently exist to stratify patients for polyphenol-based RAS therapy. This situation risks the implementation of ‘one-size-fits-all’ approaches, which can reduce efficacy and increase unnecessary exposure to treatment.

### 6.2. Patient-Centric Translational Opportunities

Precision RAS targeting optimizes patient stratification. The expression of RAS components, such as AT1R and ACE2, serves as a robust predictive biomarker for treatment response, thereby creating an opportunity to advance beyond non-selective therapy and deliver personalized care. This approach facilitates the development of RAS subtype-specific treatment algorithms. Clinical data [122] indicate that patients with AT1R-high TNBC who received losartan, an ARB, experienced a 35% reduction in recurrence risk and a 2.8-year extension in median DFS compared to non-users. In contrast, patients with AT1R-low expression did not derive any benefit from this treatment. Extending this finding to polyphenols, AT1R-high TNBC patients could be prioritized for resveratrol (500 mg/day) or quercetin, as quercetin binds to AT1R with high affinity [116]. Similarly, patients with high ACE expression in renal cell carcinoma may benefit more from specific polyphenols, which exhibit strong ACE inhibitory activity, such as rosmarinic acid (IC_50_ = 0.05 μmol/L) [78]. Combining these polyphenols with low-dose ACE inhibitors can prevent ineffective therapy, thereby directly improving patients’ chances of disease control. Furthermore, this approach supports the integration of point-of-care RAS testing, as rapid detection of RAS components is feasible. For instance, AT1R expression can be detected through serum biomarkers or immunohistochemistry, which could facilitate real-time treatment adjustments. In the context of CRC, pre-treatment testing for AT1R could identify patients most likely to benefit from combination therapy with EGCG and losartan, minimizing delays in initiating effective treatment and improving prognosis. This aligns with the findings of Reference [44], which associates high AT1R expression with reduced relapse-free survival in CRC, thereby underscoring the necessity for targeted patient selection.

Polyphenol-based combinations have been shown to reduce treatment toxicity. Natural polyphenols exhibit low toxicity even at high doses; for instance, EGCG remains non-toxic at 1000 mg/day [123]. Furthermore, these compounds share RAS-modulating mechanisms with synthetic RASi, suggesting that polyphenols may provide a viable strategy for mitigating treatment-related AEs. Such AEs frequently compromise patients’ quality of life. Notably, polyphenols can serve as alternatives to high-toxicity adjuvants. In CRC, relevant data from Reference [119] indicate that the combination of losartan and vitamin D reduces tumor number by 42%. However, high doses of vitamin D (≥10,000 IU/day) pose a risk of hypercalcemia, a common AE that can lead to fatigue and renal dysfunction. Substituting vitamin D with EGCG (1000 mg/day) effectively addresses this concern while maintaining synergistic RAS inhibition through AT1R blockade and ACE2 upregulation [90]. This substitution also mitigates the risk of hypercalcemia. Similarly, polyphenols can mitigate AEs related to RASi. Hypotension induced by losartan represents a significant barrier to long-term use. The combination of losartan with resveratrol has been shown to reduce this hypotension. Resveratrol effectively balances the dual-axis activity of the renin–angiotensin system, without exacerbating drops in blood pressure, thereby enabling patients to tolerate the therapy over extended periods. Furthermore, polyphenols have demonstrated the potential to alleviate chemotherapy-induced side effects. Preclinical data (refer to Section 3.4) provide support for this assertion, indicating that polyphenols can reduce oxidative stress and inflammation by inhibiting the RAS-NOX pathway. In clinical practice, polyphenols may relieve specific chemotherapy-induced AEs; for instance, they could mitigate neuropathy, a common side effect in CRC and breast cancer patients. Additionally, polyphenols may reduce cardiotoxicity associated with anthracyclines. Notably, resveratrol has been shown to upregulate cardiac ACE2 expression [82], which offers protection against anthracycline-induced myocardial damage and helps patients maintain their physical function. Consequently, this enables patients to complete their full treatment course, addressing a critical unmet clinical need. Currently, 30–40% of patients discontinue chemotherapy prematurely due to AEs, underscoring the significant clinical value of polyphenols in mitigating treatment-related toxicity.

Interdisciplinary technologies have the potential to overcome barriers associated with RAS targeting. Polyphenols, while promising, face challenges such as low bioavailability. However, these challenges can be addressed through interdisciplinary innovations, thereby unlocking opportunities to expand effective treatment options for patients with limited therapies. For instance, the development of RAS-targeted nanodelivery systems is a viable approach. PLGA nanoparticles can be loaded with compounds like EGCG or resveratrol [125,126], enhancing polyphenol accumulation in RAS-active tumors, such as AT1R-high melanoma, by a factor of 15. This improvement not only boosts treatment efficacy but also increases the ORR from 45% to 68% in BRAF-mutant melanoma [121]. Furthermore, it reduces off-target exposure, minimizing the risk of potential hepatotoxicity, which, although rare, is a serious adverse event associated with high-dose EGCG [123]. For patients with metastatic melanoma, this nanotechnology could render polyphenols a viable alternative to high-toxicity targeted therapies, such as BRAF inhibitors, which have a 50% incidence of cutaneous AEs. Additionally, combining organoid models with RAS research presents another innovative strategy. Patient-derived tumor organoids (PDOs) can be engineered to express RAS components; for instance, ACE-high CRC organoids exemplify this approach. These engineered PDOs can be utilized to preclinically test combinations of polyphenols and RASi. Taking CRC PDOs with high ACE expression as an example, they could be employed to validate the efficacy of rosmarinic acid in conjunction with ACE inhibitors. This strategy aids in identifying effective regimens tailored to individual patients before clinical administration, thereby reducing the risks associated with trial-and-error treatment approaches, saving patients from unnecessary AEs such as ACE inhibitor-induced cough, and improving treatment response rates.

RAS regulation enhances the response to immunotherapy. Section 5.2 (Ref. [120]) and Section 3.4 highlight two core effects of RAS inhibition. First, it normalizes the tumor microenvironment by increasing CD8+ T cell infiltration. Second, it enhances the efficacy of immunotherapy, creating a transformative opportunity that can extend survival for patients with immunotherapy-refractory tumors. Polyphenols play a crucial role in converting ‘cold’ tumors into ‘hot’ tumors. In NSCLC, EGFR-mutant tumors are typically ‘immunologically cold,’ characterized by low T cell infiltration and resistance to immune checkpoint inhibitors (ICIs). A polyphenol cocktail (EGCG + resveratrol + quercetin) can regulate RAS-related axes by upregulating ACE2, an anti-tumor axis component, and downregulating AT1R, a pro-tumor axis component. This regulation normalizes tumor vasculature and increases CD8+ T cell infiltration (Section 3.4). Combining this cocktail with anti-PD-1 antibodies may improve treatment outcomes, potentially raising the ORR from 23% (with ICI monotherapy [57]) to 38% and extending OS, with a hazard ratio (HR) of 0.71 for OS in EGFR-mutant NSCLC [120]. This provides new hope for patients with limited treatment options. Additionally, polyphenols can mitigate immune-related AEs associated with ICIs by inhibiting the RAS pathway to modulate inflammation (Section 4.1). This modulation may alleviate ICI-induced colitis or pneumonitis, which are common irAEs that often lead to treatment discontinuation. Curcumin, a polyphenol with anti-inflammatory properties, exemplifies this potential. It inhibits NF-κB activation downstream of AT1R (Section 3.4), thereby reducing ICI-related gut or lung inflammation and enabling patients to continue life-prolonging immunotherapy without the need for dose reductions, directly improving survival outcomes. Polyphenols can also address key existing challenges; for instance, nanodelivery technology can enhance their low bioavailability, while point-of-care testing can resolve the lack of relevant biomarkers. Thus, polyphenols have the potential to serve as low-toxicity adjuvants that specifically target the RAS pathway. These adjuvants not only enhance treatment efficacy but also maintain the quality of life for cancer patients. Future research should prioritize these opportunities to translate preclinical RAS-polyphenol research data into clinically meaningful therapies.

### 6.3. Polyphenol-Rich Dietary Therapy

Epidemiological evidence supports the hypothesis that populations consuming diets naturally rich in polyphenols exhibit lower incidence rates of several cancers discussed in our review. A large prospective cohort study within the European Prospective Investigation into Cancer and Nutrition (EPIC) found an inverse association between total polyphenol intake and overall cancer mortality, particularly for digestive tract cancers [127]. For instance, the traditional Mediterranean diet—characterized by high polyphenol intake and abundant in olives, red wine, fruits, vegetables, tea, and herbs—has been consistently associated with reduced risks of colorectal (RR 0.82), breast (RR 0.92), liver (RR 0.58), and gastrointestinal cancers (RR 0.72) [128]. Data from the Italian Cancer Registry Association in 2015 indicated that the prevalence of colorectal cancer in southern Italy (437 cases), where the Mediterranean diet rich in plant polyphenols is predominant, was significantly lower than in the northwest (764 cases) and northeast (775 cases) (AIRTUM Registry report on cancer. The numbers of cancer in Italy. 2015. Available at: http://www.registri-tumori.it/cms/it/node/3993 “URL (accessed on 25th October 2025)”). Similarly, Asian populations with high consumption of green tea (rich in tea polyphenols), soy (rich in isoflavones), and other plant-based foods exhibit lower age-standardized incidence rates of prostate and breast cancers compared to Western countries [129]. Meta-analyses have confirmed that the consumption of green tea is associated with reduced risks of several cancers, including oral cancer (RR = 0.798) [130], lung cancer in women (RR = 0.78) [131], and colon cancer (OR = 0.82) [132], with the most pronounced effects observed in intervention studies at a daily intake of 3–4 cups of green tea. This highlights that the potential impact of contemporary diets on cancer may operate through the mechanism of cumulative, low-dose exposure. Although polyphenols alone may have limited effects, when combined with conventional therapies, physicians can recommend practical dietary modifications for patients, such as daily consumption of 5 servings of vegetables and fruit [133] or 3 cups of green tea [134]. These evidence-based suggestions may provide additional protective benefits as adjuvant therapy.

## 7. Conclusions

Natural polyphenols represent a promising class of RAS-modulating agents with significant potential in cancer prevention and therapy. By simultaneously inhibiting the pro-tumor ACE/AngII/AT1R axis and enhancing the anti-tumor ACE2/Ang(1-7)/MasR axis, polyphenols restore the balance of RAS and suppress key oncogenic pathways. Their capacity to modify thiol-containing transmembrane proteins further amplifies their anti-cancer effects. However, limitations such as low bioavailability and insufficient clinical validation remain major barriers. Future research should focus on nanodelivery technologies, biomarker-guided patient stratification, and well-designed clinical trials to translate preclinical findings into effective, low-toxicity adjuvant therapies. Integrating polyphenol-rich diets or supplements with conventional treatments may provide a synergistic approach to improving cancer outcomes.

## Figures and Tables

**Table 1:** Illustration of natural polyphenol compounds, extracts, and their derivatives exhibiting ACE-inhibitory properties.

Categories of Polyphenols	Representative Compound	IC_50_/Inhibition Ratio	Reference
Flavonoid	3, 7-dihydroxyflavone	IC_50_ = 0.04 μmol/L	[78]
	Fisetin	IC_50_ = 0.08 μmol/L	[78]
	Luteolin	IC_50_ = 7.1 μmol/L	[78]
	Quercetin	IC_50_ = 30.3 μM	[113]
	Eupatorin	IC_50_ = 15.35 ± 4.49 µg/mL	[111]
	Sinensetin	IC_50_ = 29.5 ± 0.3 µg/mL	[111]
	3′-hydroxy-5,6,7,4′-tetramethoxyflavone	IC_50_ = 27.9 ± 2.4 µg/mL	[111]
Flavonoid derivative/flavonoid glycosides	Acteoside	IC_50_ = 0.36 μmol/L	[78]
	Isoacteoside	IC_50_ = 0.46 μmol/L	[78]
	Rutin	IC_50_ = 0.45 μmol/L	[78]
	Miquelianin	32.41% (100 ng/mL)	[114]
	Myricitrin	31.66% (100 ng/mL)	[114]
	Myricetin glycoside	IC_50_ = 10.2–14.5 μM	[113]
Phenolic acid	Rosmarinic acid	IC_50_ = 0.05 μmol/L	[78]
	Ethyl caffeate	55.19% (10 μg/mL)	[81]
	Caffeic acid	IC_50_ = 2.10 mM	[81]
	Ferulic acid	IC_50_ = 4.40 mM	[81]
Phenolic acid derivative	3-feruloylquinic acid	26.32% (100 ng/mL)	[114]
	Rosmarinic acid	IC_50_ = 18.8 ± 0.2 µg/mL	[111]
	Geraniin	IC_50_ = 13.22 uM	[112]
Anthocyanin	Delphinidin-3-O-sambur diglycoside	IC_50_ = 0.14 μmol/L	[78]
	Cyanidin-3-o-sambu diglycoside	IC_50_ = 0.11 μmol/L	[78]
Other phenolic derivative	(+)-trans-Decursidinol	IC_50_ = 0.00468 mM	[81]
	Butein	IC_50_ = 8.44 μmol/L	[78]
Polyphenol complexes	Coriandrum sativum flavonoid-rich fraction (containing pinocembrin, apigenin)	IC_50_ = 28.91 ± 13.42 μg/mL	[114]
Polyphenol extracts	Kiwifruit aqueous extracts	47.71% (2.8 mg/g Dry weight)	[115]
	Ocimum basilicum leaves extracts (containing Rutin, quercetin, and quercitrin, caffeic, chlorogenic, and gallic acids)	IC_50_ = 64.99 µg/mL	[112]
	O. Gratissimum leaves extracts (containing Rutin, quercitrin, luteolin; ellagic, chlorogenic acids)	IC_50_ = 29.44 µg/mL	[112]
	Mesembryanthemum crystallinum extracts (containing apigenin, diosmin and luteolin)	90.5% (1 mg/mL)	[81]

## Data Availability

Data derived from public domain resources. The data presented in this study are available in reference numbers [78,81,111,112,113,114,118,119,120,121,122], etc.

## Abbreviations

The following abbreviations are used in this manuscript:

RAS	renin–angiotensin system
AngII	angiotensin II
ACE	angiotensin-converting enzyme
AT1R	angiotensin type 1 receptor
AT2R	angiotensin type 2 receptor
RASi	RAS inhibitors
ACE2	angiotensin-converting enzyme 2
Ang(1-7)	angiotensin 1-7
ACEis	ACE inhibitors
ARBs	angiotensin receptor blockers
AGT	angiotensin
CC-RCC	clear cell renal cell carcinoma
CRC	colorectal cancer CRC
EGCG	epigallocatechin gallate
EAOP	EGCG autoxidation products
P-gp	P-glycoprotein
ROS	reactive oxygen species
NO	nitric oxide
NOX	NADPH oxidase
(P)RR	(Pro)renin receptor
PFS	progression-free survival
DKK3	dickkopf-3
QSOX1	Quiescin Sulfhydryl Oxidase 1
LPS	lipopolysaccharide
TNBC	triple-negative breast cancer
CSM	cancer-specific mortality
OM	overall mortality
VEGF	vascular endothelial growth factor
NSCLC	non-small cell lung cancer
ORR	objective response rate
OS	overall survival
ICI	immune checkpoint inhibitors
EPIC	the European Prospective Investigation into Cancer and Nutrition
DFS	disease-free survival
AEs	adverse events
ADAM17	a disintegrin and metalloproteinase 17
MAPK	mitogen-activated protein kinase
NF-κB	NF-kappaB
STATs	signal transducers and activators of transcription
